# Interaction of *Leptospira* Elongation Factor Tu with Plasminogen and Complement Factor H: A Metabolic Leptospiral Protein with Moonlighting Activities

**DOI:** 10.1371/journal.pone.0081818

**Published:** 2013-11-27

**Authors:** Danielly G. Wolff, Mónica M. Castiblanco-Valencia, Cecília M. Abe, Denize Monaris, Zenaide M. Morais, Gisele O. Souza, Sílvio A. Vasconcellos, Lourdes Isaac, Patrícia A. E. Abreu, Angela S. Barbosa

**Affiliations:** 1 Laboratório de Bacteriologia, Instituto Butantan, São Paulo, Brasil; 2 Departamento de Imunologia, Instituto de Ciências Biomédicas, Universidade de São Paulo, São Paulo, Brasil; 3 Laboratório de Biologia Celular, Instituto Butantan, São Paulo, Brasil; 4 Faculdade de Medicina Veterinária e Zootecnia, Universidade de São Paulo, São Paulo, Brasil; University of São Paulo School of Medicine, Brazil

## Abstract

The elongation factor Tu (EF-Tu), an abundant bacterial protein involved in protein synthesis, has been shown to display moonlighting activities. Known to perform more than one function at different times or in different places, it is found in several subcellular locations in a single organism, and may serve as a virulence factor in a range of important human pathogens. Here we demonstrate that *Leptospira* EF-Tu is surface-exposed and performs additional roles as a cell-surface receptor for host plasma proteins. It binds plasminogen in a dose-dependent manner, and lysine residues are critical for this interaction. Bound plasminogen is converted to active plasmin, which, in turn, is able to cleave the natural substrates C3b and fibrinogen. *Leptospira* EF-Tu also acquires the complement regulator Factor H (FH). FH bound to immobilized EF-Tu displays cofactor activity, mediating C3b degradation by Factor I (FI). In this manner, EF-Tu may contribute to leptospiral tissue invasion and complement inactivation. To our knowledge, this is the first description of a leptospiral protein exhibiting moonlighting activities.

## Introduction

Pathogenic microorganisms are able to penetrate and colonize host tissues by establishing complex interactions with host molecules. Surface proteins mediate binding of microbial pathogens to an array of host targets such as cell receptors, extracellular matrix components, coagulation cascade molecules and complement regulators. This process enables pathogens to disseminate to multiple organs and to evade host´s innate immune response. 

Recent reports have attributed unexpected functions to cytosolic bacterial proteins normally involved in key metabolic processes or in the cell stress response (reviewed in 1). Known as “moonlighting” proteins, they execute multiple and unrelated functions, and are found in several subcellular locations in a single organism. By performing diverse cellular functions related to adhesion, invasion and modulation of the host immune system, this intriguing class of proteins may contribute to virulence of important pathogens [1,2]. Enzymes of the glycolytic pathway and of other metabolic pathways such as the glyoxylate cycle, usually expressed at high levels, are good examples of proteins that moonlight in bacteria. Molecular chaperones and proteins involved in protein synthesis may also perform multiple functions in some microorganisms [1]. 

The elongation factor Tu (EF-Tu) is an abundant bacterial protein identified as a carrier of aa-tRNA to the ribosome, a function associated with hydrolysis of the bound GTP [3]. Besides its role in protein synthesis, a panoply of functions has been proposed for EF-Tu, including chaperone activity [4] and catalyzation of protein disulfide formation, reduction, and isomerization (refolding of randomly oxidized RNase) [5]. EF-Tu has been described to be cell surface associated in several prokaryotes, contributing to adhesion and invasion processes. In *Mycoplasma pneumonia* EF-Tu mediates binding to fibronectin [6], and in *Lactobacillus johnsonii* it is implicated in the attachment to human intestinal cells and mucins [7]. Interaction of EF-Tu with the human complement regulator FH and with plasminogen has been reported for *Pseudomonas aeruginosa*, thus facilitating bacterial immune evasion and tissue invasion [8].

Spirochetes from the genus *Leptospira* are the aetiological agent for leptospirosis, a neglected infectious disease that constitutes a major public health problem in developing countries. Once inside the host, pathogenic leptospires are able to spread and colonize multiple organs. Invasiveness is attributed to their ability to circumvent host´s innate immune response and adhere to host cells and extracellular matrices (reviewed in 9). Leptospires are also capable of binding and activating plasminogen on their surfaces [10,11]. Once converted to its active serine protease form (plasmin), this key enzyme of the coagulation system is crucial for blood clot resolution by dissolving fibrin polymers. Plasmin also degrades extracellular matrix macromolecules including the glycoproteins fibronectin, laminin, and elastin as well as proteoglycans [12]. Recently, plasmin has been characterized as a complement inhibitor by cleaving the key proteins C3b and C5 [13]. The surface protein E from *Haemophilus influenzae* acquires human plasminogen that, once converted to plasmin, allows the bacterium to control complement by degrading C3b [14].

Given the role of EF-Tu in bacterial adhesion, invasion and immune evasion, our goal in the present study was to characterize this multitask protein in *Leptospira*. We demonstrate that leptospiral EF-Tu is highly conserved among diverse species and is surface localized. Moreover, it is a FH- and a plasminogen-binding protein. Bound to EF-Tu, plasminogen is converted to plasmin, which in turn cleaves the central human complement protein C3b as well as the coagulation cascade molecule fibrinogen. In this manner, EF-Tu may aid leptospires to disseminate throughout host tissues and to evade innate immunity. 

## Materials and Methods

### Ethics Committee Approval

Animals were supplied with food and water *ad libitum* and experimental protocols were previously approved by the Ethical Committee for Animal Research of the Faculdade de Medicina Veterinária and Zootecnia, Universidade de São Paulo, São Paulo, Brazil, under the license number 2385/2011.

### Bacterial strains and plasmids

*Leptospira biflexa* serovar Patoc strain Patoc I, *Leptospira noguchii* serovar Panama strain CZ 214K, *Leptospira borgpetersenii* serovar Javanica strain Veldrat Batavia 46, *Leptospira borgpetersenii* serovar Tarassovi strain 17, *Leptospira kirschneri* serovar Cynopteri strain 3522C, *Leptospira interrogans* serovar Copenhageni strain 10A, *Leptospira interrogans* serovar Copenhageni strain L1-130, *Leptospira interrogans* serovar Pomona strain Pomona, and *Leptospira santarosai* serovar Shermani strain 1342K were used in the assays. Bacteria were cultured at 29°C under aerobic conditions as previously described [15]. *Escherichia coli* DH5α was used as the cloning host strain and E. coli BL21 (DE3) was used as the host strain for the expression of the recombinant proteins, using the T7 promoter based expression plasmid pAE [16]. 

### Purified proteins, sera and antibodies

All macromolecules from the extracellular matrix (ECM) were purchased from Sigma-Aldrich. Laminin-1 and collagen Type IV were derived from the basement membrane of Engelbreth-Holm-Swarm mouse sarcoma, cellular fibronectin was derived from human foreskin fibroblasts, plasma fibronectin was isolated from human plasma, collagen Type I was isolated from rat tail, and elastin from human aorta. Fibrinogen and plasminogen were isolated from human plasma. Human FH, C3b and FI were purchased from Complement Technology. Normal human sera (NHS) were obtained from healthy donors. The sera were pooled, and stored in aliquots at -80°C until use. Goat anti-human FH was purchased from Quidel, goat anti-human C3 polyclonal antibody was purchased from Complement Technology and secondary peroxidase-conjugated antibodies from Sigma-Aldrich. Mouse monoclonal anti-human fibrinogen was purchased from BD Biosciences. 

### Cloning, expression, purification of recombinant proteins and generation of antiserum

The *tuf* gene (LIC12875) was amplified by PCR from genomic DNA of *L. interrogans* serovar Copenhageni strain 10A using the primers: F:CGCTCGAGGCTAAAGAAAAG / R:GCGAAGCTTTTACTCAGTG. PCR fragments were cloned into pGEM T-Easy vector (Promega) and transformed into *E. coli* DH5α. Following digestion with restriction enzymes *Xho* I and *Hind* III, fragments were subcloned into the *E. coli* expression vector pAE. Expression and purification of the resulting 6XHis-tagged recombinant protein were performed as previously described [17]. The protein was purified from the supernatant, and also from the insoluble pellet by nickel affinity chromatography. LIC10301, LipL32 and LigBC were expressed and purified as previously described [15,18]. The pAE-*lipl32* construct was kindly provided by Dr. Paulo Lee Ho (Instituto Butantan, São Paulo, Brazil). Antisera were produced in mice [17]. 

### Surface immunofluorescence assay

This assay was performed as previously described [19] with slight modifications. Briefly, a 250 µL suspension of 1 x 10^8^ *L. interrogans* serovar Copenhageni strain L1-130 was added to each well of Lab-Tek eight-well chamber slides (Nalge Nunc). Following a 80 min-incubation at 30°C, unbound leptospires were carefully removed, and adhered bacteria were fixed with 2% paraformaldehyde in PBS. Slides were incubated for 90 min at 30°C in blocking buffer (Difco Leptospira Enrichment EMJH, BD). Immune and pre-immune sera (1:50) were diluted in blocking buffer and slides were incubated for 60 min at 30°C. After three washes with PBS, Alexa Fluor 488-labeled goat anti-mouse IgG (Invitrogen / Molecular Probes) diluted 1:500 in blocking buffer was added to the slides. Incubation proceeded for 45 min at 30°C, and the slides were washed twice with PBS and once with sterile water. The chambers were removed and the slides were mounted with Vectashield medium containing propidium iodide (Vector Laboratories). Images were collected using a LSM 510 (Zeiss) laser scanning confocal microscope.

### Immunogold labeling and negative staining

*L. interrogans* serovar Copenhageni strain L1-130 fixed with 0.3% glutaraldehyde in PBS were firstly blocked with PBS containing 0.2% bovine serum albumin (PBS/BSA) for 30 min, and then incubated for 1h at room temperature with anti-EF-Tu or preimmune serum (negative control) diluted 1:10 in PBS/BSA. After washings with PBS, preparations were incubated with goat anti-mouse antibody labeled with 10 nm colloidal gold particles (Sigma-Aldrich, Co., USA) diluted 1:5 in PBS/BSA for another hour, at room temperature. After washings with PBS and distilled water, preparations were negatively stained with 2% uranyl acetate, applied onto Formvar-coated nickel grids for 2 min, air dried, and observed under TEM (LEO 906E - Leica Microsystems BmgH, Germany) at 80 kV.

### Binding of EF-Tu to ECM and coagulation cascade molecules

EF-Tu attachment to individual macromolecules was analyzed by an ELISA-based assay according to a previously published protocol [17]. LigBC and LIC10301 were used as positive and negative controls, respectively. Bound proteins were detected with specific mouse antisera (1:10000). To determine the role of lysines in EF-Tu plasminogen interactions, ELISA plate wells were coated with recombinant EF-Tu (10 µg/mL). The same protocol mentioned above was followed except that ε-aminocaproic acid (0 - 10 mM) was added with plasminogen (10 µg/mL) to EF-Tu-coated wells. Bound plasminogen was detected with a mouse monoclonal antibody (Sigma-Aldrich) at a 1:500 dilution followed by peroxidase-conjugated anti-mouse IgG (Sigma-Aldrich) at a 1:5000 dilution. Student´s two-tailed *t* test was used for statistical analyses. A P value less than 0.05 was considered statistically significant.

### Plasmin activity after plasminogen activation

Microtiter plate wells were coated with recombinant proteins (10 µg/mL). After blocking with 3% BSA diluted in PBS, plasminogen (20 µg/mL) was added and incubation proceeded for 1 h at 37° C. Unbound plasminogen was removed by washing wells three times with PBS-0.05% Tween, pH 7.4 (PBS-T), and then human urokinase plasminogen activator (uPA) (3 U) and the chromogenic substrate D-valyl-leucyl-lysine-ρ-nitroanilide dihydrochloride (25 µg/well) dissolved in PBS were added. The plates were incubated at 37° C and absorbance at 405 nm was read after 24 h.

### Fibrinogen and C3b degradation assay

Recombinant proteins (10 µg/mL) were immobilized onto microtiter plate wells. After blocking with 3% BSA diluted in PBS, plasminogen (20 µg/mL) was added and incubation proceeded for 1 h at 37° C. Wells were washed with PBS-T and human fibrinogen (500 ng, plasminogen depleted; Calbiochem) or human C3b (500 ng) together with plasminogen activator uPA (3 U) were added. Reaction mixtures were incubated at 37° C for the indicated time points, and were then separated by SDS-PAGE and transferred to nitrocellulose membranes. The degradation products were detected by Western blotting using a mouse monoclonal anti-human fibrinogen α-chain (1:3000) or a goat polyclonal anti-human C3 (1:10000) and the corresponding secondary horseradish phosphatase-conjugated antibodies. Membranes were developed with SuperSignal West Pico (Pierce).

### Interaction of EF-Tu with FH by ligand affinity blotting

Purified recombinant proteins were subjected to 10% SDS–PAGE under nonreducing conditions and transferred to nitrocellulose membranes. The membranes were incubated for 90 min with 7% normal human serum as a source of FH diluted in PBS. After washing, the membranes were incubated with polyclonal goat antibodies recognizing human FH (1:10000), followed by peroxidase-conjugated secondary antibodies (1:10000). Positive signals were detected by enhanced chemiluminescence (West Pico, Pierce). LigBC and LIC10301 were used as positive and negative controls respectively [18]. 

### Cofactor assay

Cofactor activity of FH bound to EF-Tu was analyzed by measuring FI-mediated cleavage of C3b essentially as described by 18. LigBC and LIC10301 were used as positive and negative controls respectively [18]. 

### Immunoblot analysis

*Leptospira* extracts were fractionated on a 12% SDS–PAGE and transferred to nitrocellulose membranes. Nonspecific binding sites were blocked by using 10% (wt/vol) dried milk in PBST overnight at 4° C. After washing three times with PBS-T, the membranes were incubated with mouse anti-EF-Tu serum (diluted 1:1000) in 5% non-fat dried milk–PBST for 60 min. Following three washes with PBST, the membranes were incubated with a secondary peroxidase-conjugated anti-mouse IgG for 60 min at room temperature at a 1:5000 dilution, washed, and revealed with ECL reagent (West Pico, Pierce).

## Results

### EF-Tu is a surface protein of *Leptospira*

**Figure 1:**
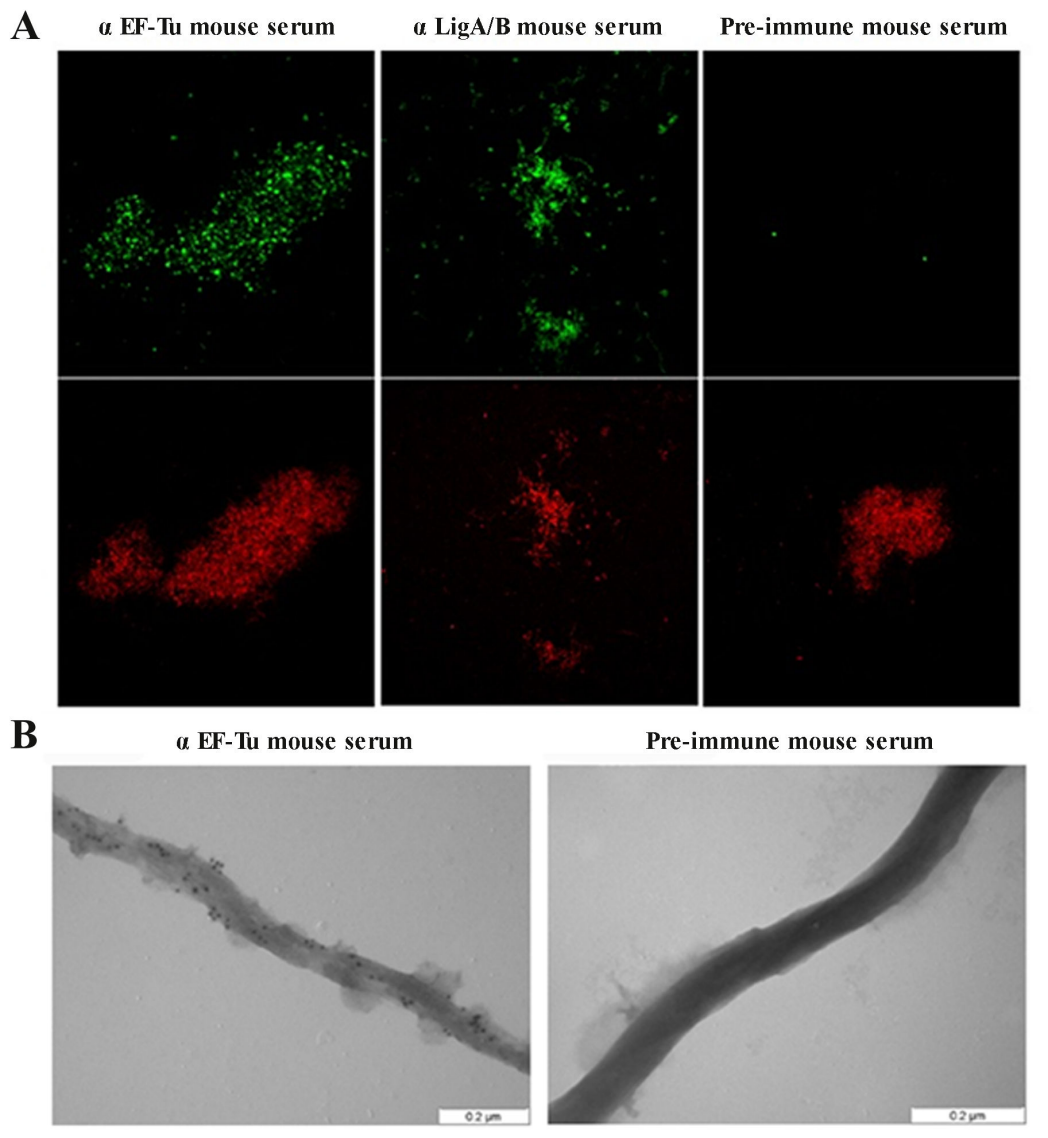
Cellular localization of EF-Tu on *Leptospira interrogans*. (**A**) Surface immunofluorescence assay was performed using confocal microscopy (Zeiss LSM-510 Meta). Intact leptospires were probed with anti-EF-Tu, anti-LigA/B or preimmune mouse serum. Alexa Fluor 488 conjugated goat anti-mouse IgG was used to detect bound antibodies. A iodide propidium counterstain (low panels) was used to demonstrate the presence of leptospires. (**B**) Immunoelectron microscopy analysis. Leptospires were incubated with anti-EF-Tu or pre-immune mouse serum, followed by treatment with colloidal-gold-conjugated anti-mouse IgG. Analysis was performed using an electron transmission microscope (LEO 906E - Leica Microsystems BmgH, Germany).

Proteins supposed to have multiple functions may display more than one subcellular localization. In order to assess if EF-Tu is associated to the leptospiral membrane, we performed immunofluorescence assays with intact bacteria. Leptospires were cultured until they reached a density of 1 x 10^8^ cells/mL and were then added to the slides. To avoid outer membrane disruption, the initial centrifugation step, normally used to harvest and concentrate the bacteria [19], was abolished. Our results revealed that EF-Tu was recognized by anti-EF-Tu mouse serum (Figure 1A). Positive control experiments were performed with antibodies recognizing the conserved N-terminal portion shared by LigA and LigB (anti-LigA/B mouse serum), known to be surface-exposed proteins [20], and preimmune mouse serum was used as a negative control (Figure 1A). EF-Tu surface localization was further assessed by immunoelectron microscopy. Immunogold labeling of *L. interrogans* cells was consistently observed with antiserum against EF-Tu, while organisms incubated with preimmune serum did not present bound colloidal-gold particles (Figure 1B). 

### EF-Tu interacts with ECM components and coagulation cascade molecules

**Figure 2:**
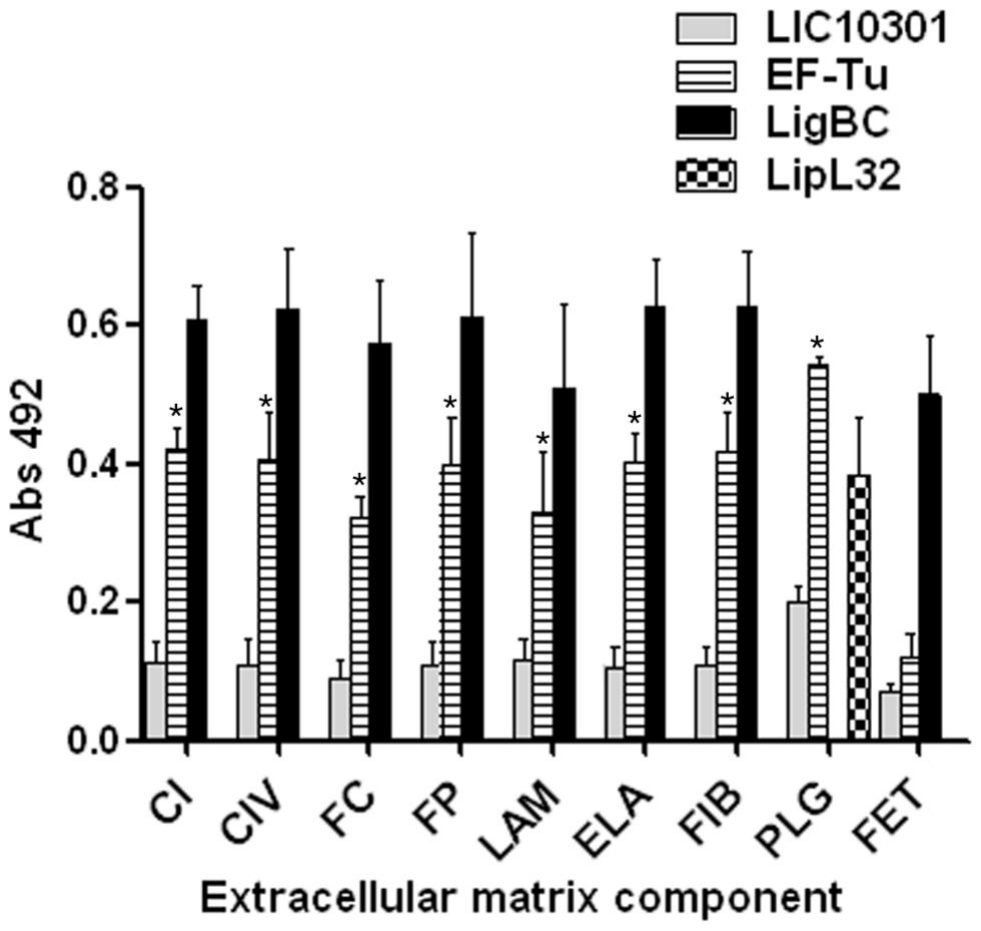
Binding of EF-Tu to ECM components. Wells were coated with 10 μg/mL of collagen type I (CI), collagen type IV (CIV), cellular fibronectin (FC), plasma fibronectin (FP), laminin (LAM), elastin (ELA), fibrinogen (FIB), plasminogen (PLG) and the control protein fetuin (FET). Recombinant protein attachment to those ECM macromolecules was assessed by ELISA. One microgram of recombinant EF-Tu protein was added per well. LigBC and LIC10301 were included as positive and negative controls, respectively. LipL32 was included as a positive control for plasminogen [34]. Bound proteins were detected using mouse specific antisera to the recombinant proteins, followed by peroxidase-conjugated secondary antibodies. Each point represents the mean absorbance value at 492 nm ± the standard deviation of three independent experiments, each performed in duplicate. Binding of EF-Tu to each ECM component was compared to the binding of LIC10301 to these molecules by the two-tailed t test (* *p* < 0.05).

Once EF-Tu is associated to the leptospiral membrane, we next investigated its ability to interact with host molecules such as ECM and coagulation cascade components. According to our ELISA binding assays, EF-Tu interacted will all ECM macromolecules tested. Binding to fibrinogen and plasminogen was also observed. EF-Tu did not bind to fetuin, included as a highly glycosylated attachment-negative control protein (Figure 2). Interestingly, LigBC, our positive control previously shown to bind multiple ECM macromolecules [21-28], strongly interacted with fetuin, but no specific binding to the target molecules was detected with the negative control protein LIC10301 (Figure 2).

### Role of lysine residues in EF-Tu binding to plasminogen

**Figure 3:**
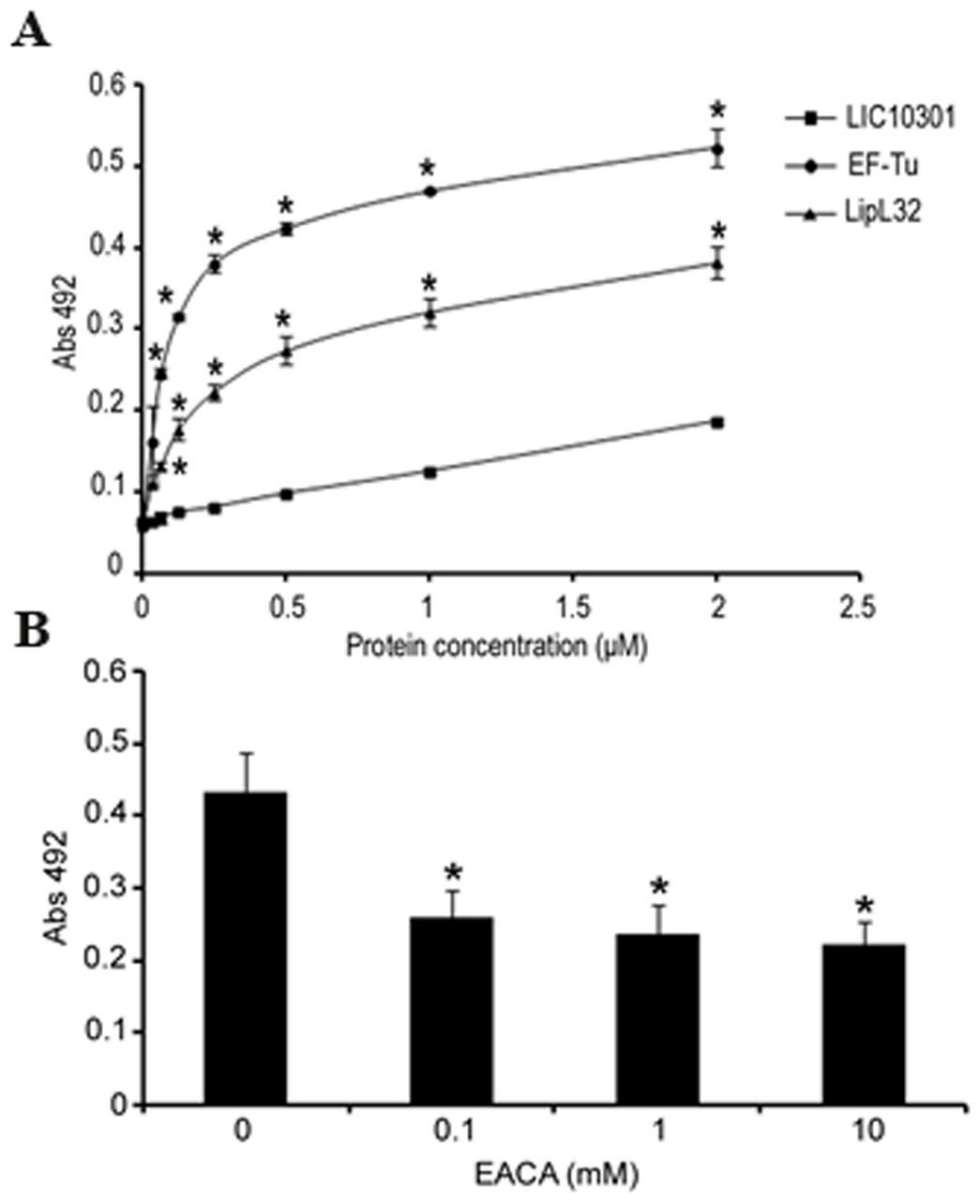
Binding of leptospiral EF-Tu to human plasminogen. (**A**) Binding of EF-Tu to plasminogen as a function of protein concentration by ELISA. EF-Tu and the positive and negative control proteins LipL32 and LIC10301 (0 - 2 μM) were allowed to interact with immobilized plasminogen (10 μg/mL), and were detected using specific antisera, followed by peroxidase-conjugated secondary antibodies. (**B**) Role of lysines in EF-Tu/plasminogen interaction. Plasminogen (10 μg/mL) was added to EF-Tu-coated wells in the presence (0.1 - 10 mM) or absence of ε-aminocaproic acid. Bound plasminogen was detected with a specific monoclonal antibody followed by peroxidase-conjugated anti-mouse IgG. In (**A**) and (**B**) each point represents the mean absorbance value at 492 nm ± the standard deviation of three independent experiments, each performed in duplicate. (* *p* < 0.05). .

According to the results shown in Figure 2, plasminogen is one of the target host molecules for EF-Tu. To further characterize this interaction, we performed a second ELISA binding assay using increasing amounts of recombinant EF-Tu. From this assay we conclude that EF-Tu binds plasminogen in a dose-dependent and saturable manner (Figure 3A). Apparently, lysine residues are relevant to plasminogen-EF-Tu interactions as the lysine analog ε-aminocaproic acid partially inhibited plasminogen binding (Figure 3B). 

### Plasminogen bound to EF-Tu is converted to plasmin

**Figure 4:**
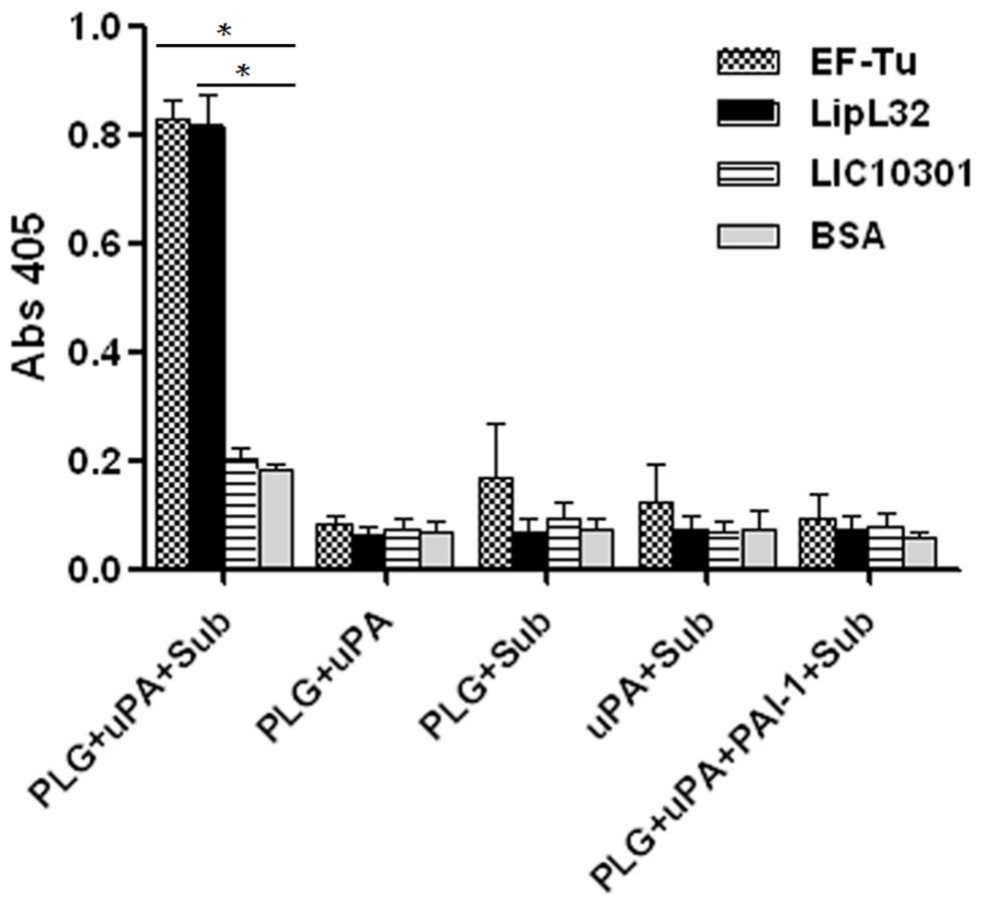
EF-Tu-bound plasminogen is converted to functionally active plasmin. Recombinant proteins or BSA (10 μg/mL), immobilized to microtiter plate wells, were incubated with plasminogen (20 μg/mL). After washing, uPA (3 U) and the chromogenic substrate D-valyl-leucyl-lysine-ρ-nitroanilide dihydrochloride (25µg/well) were added. Data represent the mean absorbance value at 405 nm ± the standard deviation of three independent experiments, each performed in duplicate. (* *p* < 0.05). .

To assess if EF-Tu-bound plasminogen could be converted to active plasmin by exogenously supplied uPA, immobilized EF-Tu was incubated with plasminogen. After extensive washing, uPA and the chromogenic substrate D-valyl-leucyl-lysine-ρ-nitroanilide dihydrochloride were added. The newly generated plasmin was able to cleave the chromogenic substrate (Figure 4).

### Plasmin bound to EF-Tu cleaves C3b and fibrinogen

**Figure 5:**
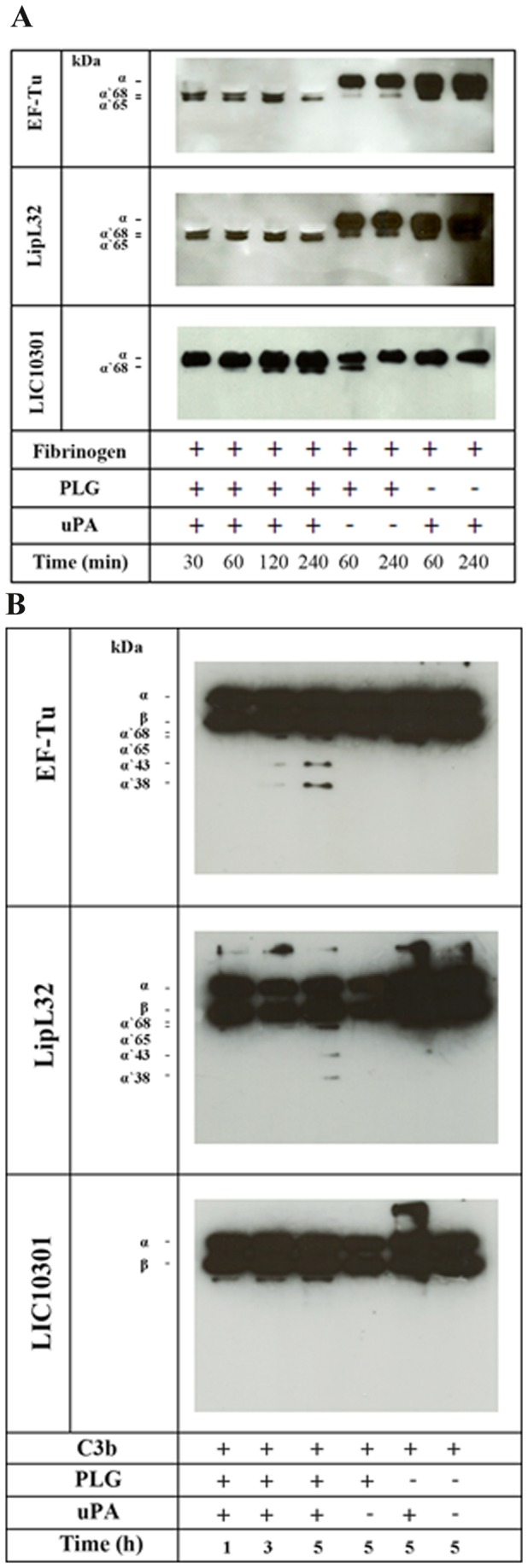
Degradation of human fibrinogen and C3b by plasmin(ogen) bound to immobilized EF-Tu. Plasminogen (20 µg/mL) was added to immobilized recombinant proteins (10 µg/mL). After washing, fibrinogen (500 ng) (**A**) or C3b (500 ng) (**B**) and uPA (3 U) were added, and incubation proceeded for the indicated time points. Samples were separated by SDS-PAGE, transferred to a nitrocellulose membrane, and probed with a mouse monoclonal antibody recognizing the fibrinogen α-chain (**A**) or a goat polyclonal anti-human C3 (**B**) followed by the corresponding secondary HRP-conjugated antibodies. Controls omitting uPA and/or plasminogen were included.

Besides its function in fibrinolysis, the serine protease plasmin plays a crucial role in degrading extracellular matrix components. It is also capable of cleaving the complement proteins such as the fragment C3b [13]. We then assayed whether EF-Tu-bound plasmin(ogen) was able to cleave the natural substrate fibrinogen and also C3b. The fibrinogen α-chain was degraded in a time-dependent manner, and cleavage was almost complete after 4 hours of incubation (Figure 5A). Proteolytic activity of EF-Tu-bound plasmin was also observed against C3b. Cleavage was time dependent, being more pronounced after 5 hours of incubation (Figure 5B). In both assays LipL32 was included as a positive control. According to our results, LipL32-bound plasmin(ogen) was able to degrade fibrinogen as efficiently as EF-Tu-bound plasmin(ogen) (Figure 5A). However, C3b cleavage was less pronounced when we used LipL32 (Figure 5B).

### EF-Tu binds FH and bound-FH retains co-factor activity

**Figure 6:**
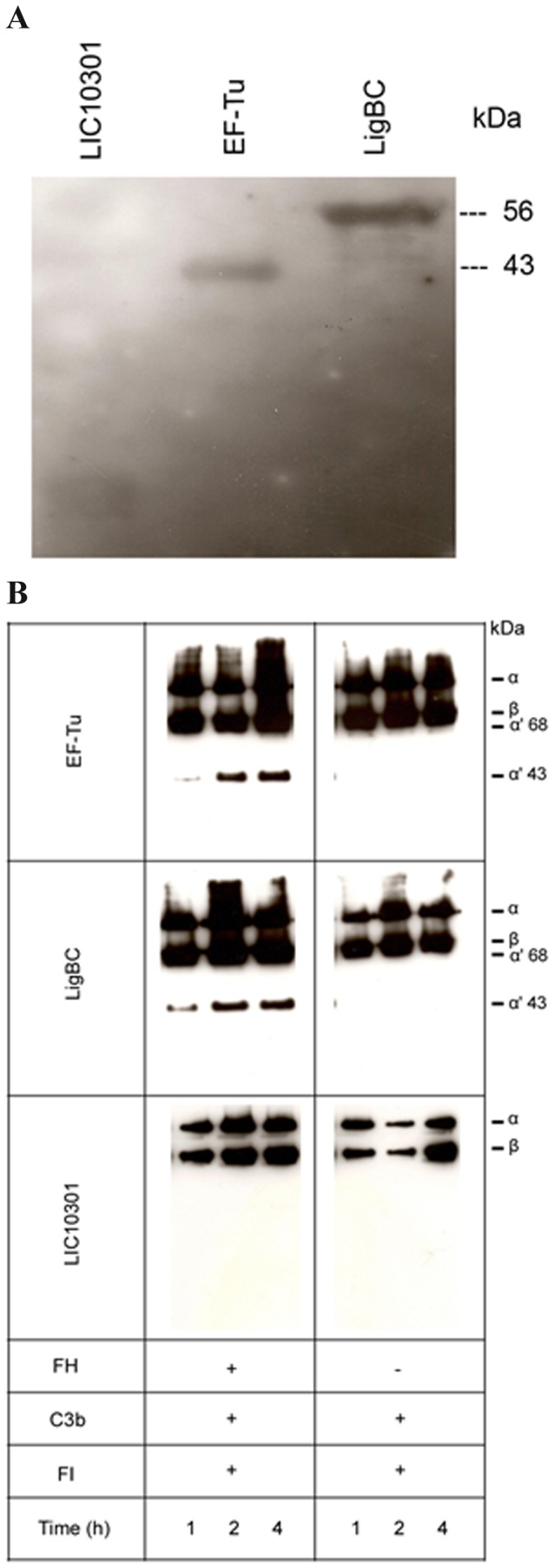
Leptospiral EF-Tu interacts with the complement regulator FH and bound-FH remains functionally active. (**A**) Purified recombinant proteins were subjected to SDS–PAGE, and transferred to a nitrocellulose membrane. The membrane was incubated with 7% NHS as a source of FH, and probed with polyclonal goat antibodies recognizing human FH, followed by secondary HRP-conjugated antibodies. LigBC (56 kDa) and LIC10301 (13 kDa) were included as positive and negative controls, respectively. (**B**) **Cofactor activity of FH bound to EF-Tu**. The recombinant proteins EF-Tu, LigBC, and LIC10301 (10 μg/mL) were immobilized on microtiter plates and incubated with purified FH (2 μg). Control reactions in which we omitted FH were also included. After washing, C3b and FI were added. The reactions were incubated for 1, 2, and 4 h at 37°C. The products were analyzed by SDS-PAGE and the cleavage fragments of C3b were detected by Western blotting with anti-human C3 polyclonal. The presence of bands of 43 and 68 kDa indicates that acquired FH was able to promote FI-mediated cleavage of C3b. LIC10301 was used as a negative control since this protein does not bind FH and LigBC was included as a positive control [18].

It has been demonstrated that EF-Tu from the Gram-negative bacterium *Pseudomonas aeruginosa* binds plasminogen and the human complement regulator FH [8]. This observation prompted us to evaluate the interaction of leptospiral EF-Tu with FH. Binding was assessed by ligand affinity blot using soluble FH from human sera. As depicted in Figure 6A, FH bound to EF-Tu. LigBC and LIC10301 were included as positive and negative controls respectively [18]. To assess the functional activity of bound-FH, EF-Tu and the control recombinant proteins were immobilized and incubated with purified FH. After washing, C3b and FI were added. The cleavage products of C3b were detected by Western blotting using anti-human C3 polyclonal antibodies. FH bound to EF-Tu retained cofactor activity, as indicated by the presence of representative C3b cleavage products (α’68 kDa and α’43 kDa). As expected, a similar profile was observed for LigBC. No cleavage products were detected for LIC10301 (Figure 6B).

### Ubiquitous distribution of EF-Tu protein in *Leptospira* spp

**Figure 7:**
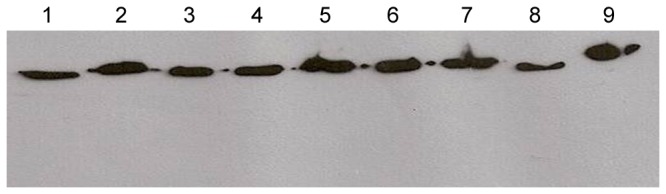
Immunoblot analysis with EF-Tu antiserum to different *Leptospira* species. Lanes contain whole-cell lysates of *L. biflexa* serovar Patoc (lane 1), *L. noguchii* serovar Panama (lane 2), *L. borgpetersenii* serovar Javanica (lane 3), *L. borgpetersenii* serovar Tarassovi (lane 4), *L. kirschneri* serovar Cynopteri (lane 5), *L. interrogans* serovar Copenhageni (lane 6), *L. interrogans* serovar Pomona (lane 7), *L. santarosai* serovar Shermani (lane 8), and recombinant EF-Tu (lane 9).

Anti-EF-Tu serum was used to screen a panel of *Leptospira* extracts. A band of 43 kDa, corresponding to the expected size of native EF-Tu, was observed in all the pathogenic serovars tested (serovars Panama, Javanica, Tarassovi, Cynopteri, Copenhageni, Pomona and Shermani), and was also observed in the non-pathogenic saprophytic *L. biflexa* serovar Patoc (Figure 7). Hence, EF-Tu is ubiquitously distributed among *Leptospira* species. Multiple sequence alignment has shown that EF-Tu from pathogenic *Leptospira* strains is highly conserved (99-100% identity at the amino acid level). *L. interrogans* Copenhageni L1-130 and *L. biflexa* Patoc1 EF-Tu share 86% sequence identity. Comparison of surface EF-Tu functions in various microorganisms indicates that this moonlighting protein interacts with host cells or molecules such as complement regulators and/or extracellular matrix proteins, and is highly conserved (Table 1).

**Table 1:** Function of surface EF-Tu in different microorganisms.

**Microorganism**	**Function (associated with surface localization)**		* % identity to *L. interrogans* Copenhageni L1-130		* % similarity to *L. interrogans* Copenhageni L1-130
***Pseudomonas aeruginosa***		Interaction whit human complement Factor H and plasminogen [8]	72%	86%	
***Francisella novicida***		Eliciting of inflammatory cytokine response in macrophages [41]	70%	85%	
***Listeria monocytogenes***		Interaction with plasminogen [31]	72%	84%	
***Mycoplasma pneumoniae***		Interaction with fibronectin [6; 42]	68%	80%	
***Lactobacillus johnsonii***		Binding to human intestinal cells and mucin [7]	66%	80%	

* Percent identity and similarity at the amino acid level was calculated using BLASTp (http://blast.ncbi.nlm.nih.gov/).

## Discussion

The Elongation Factor Tu is one of the most abundant and conserved bacterial proteins. It belongs to a class referred to as moonlighting proteins, known to perform multiple but unrelated functions that cannot be ascribed to gene fusions, splice variants or proteolytic fragments that serve different activities [29]. Along with its traditional cytoplasmic function in protein synthesis, we have demonstrated in this study that *Leptospira* EF-Tu performs additional roles as a cell-surface receptor for host plasma proteins. It remains unclear how EF-Tu, which lacks classical sorting signals, is anchored at the outer membrane of several prokaryotes, but it has been shown that this additional surface location allows interaction of these microorganisms with host cells and molecules such as fibronectin [6,30], plasminogen [8,31], FH [8], mucins and human intestinal cells [7].

Efficient colonization of target organs by pathogenic leptospires is achieved by their capacity to escape host innate immune responses [32,33] and, subsequently, through their ability to interact with host cells or with the extracellular matrix (reviewed in 9). Our immunofluorescence and immunoelectron microscopy assays indicate that *Leptospira* EF-Tu is surface localized. We then wondered whether this protein would also “moonlight”, thus contributing to leptospiral invasiveness. According to our results, EF-Tu mediates interaction with extracellular matrix and coagulation cascade molecules, including collagen I and IV, cellular and plasma fibronectin, laminin, elastin, fibrinogen and plasminogen. A proteomic approach employing total protein extracts of *L. interrogans* had already identified EF-Tu as a plasminogen-binding protein [34], what prompted us to further characterize this interaction. In agreement to previous published data, *Leptospira* EF-Tu bound human plasminogen in a dose-dependent manner. Ionic interactions do not seem to play a role in EF-Tu-plasminogen interaction, since different concentrations of NaCl did not affect binding (data not shown). Conversely, addition of ε-aminocaproic acid reduced the interaction between EF-Tu and plasminogen, thus suggesting a role for lysines in this process. Once bound to EF-Tu, plasminogen is converted to functionally active plasmin, which, in turn, is able to cleave the complement protein C3b and the fibrinogen α-chain. Interestingly, *Leptospira* EF-Tu also acquires FH, a 150-kDa plasma protein that inhibits the alternative pathway of complement by preventing binding of Factor B to C3b, accelerating decay of the C3-convertase C3bBb and acting as a cofactor for the cleavage of C3b by Factor I (FI) [35–37]. Importantly, FH is capable of acting as a cofactor when bound to EF-Tu, as indicated by the presence of C3b cleavage fragments after incubation with FI. Therefore, by interacting with plasminogen and FH, EF-Tu helps to control complement thus contributing to leptospiral immune evasion. Moreover, EF-Tu-bound plasmin(ogen) may also aid bacterial dissemination by degrading the natural substrate fibrinogen.

EF-Tu is ubiquitously distributed among leptospiral serovars, including the nonpathogenic *L. biflexa* serovar Patoc (Figure 7). In *Pseudomonas aeruginosa* EF-Tu, described as a FH- and plasminogen-binding protein, was identified in the surface protein fraction of the serum-sensitive strain SG137 [8]. The presence of EF-Tu in nonpathogenic bacteria does not warrant their survival in the host, because the majority of pathogens have generally adopted multiple evasion strategies for efficient colonization. Recent published data by our group have demonstrated that *L. interrogans* has both acquired and endogenous complement regulatory activity with regard to C3b degradation [18]. Leptospiral outer membrane proteins like LenA, LenB, LigA, LigB and LcpA have been shown to contribute to complement evasion [15,18,38,39]. Present only in pathogenic species, these receptors acquire the host fluid-phase regulators FH and/or C4b Binding Protein (C4BP), thus aiding to control complement activation at the bacterium surface. Moreover, pathogenic leptospires secrete proteases that cleave key complement proteins of the three pathways, thus conferring additional survival advantage in the host [Fraga et al., unpublished data].

EF-Tu has been described as a potential vaccine antigen against *Burkholderia* infection [40]. Given the wide distribution of this protein among diverse *Leptospira* species, its usefulness as a subunit vaccine candidate against pathogenic *Leptospira* was assessed. EF-Tu did not elicit protection in hamsters challenged with lethal doses of *L. interrogans* serovar Copenhageni L1-130 (data not shown). 

In conclusion, we identified EF-Tu as a leptospiral moonlighting protein. When displayed on the bacterium surface, EF-Tu binds multiple host effector proteins, thus contributing to tissue invasion and complement inactivation. To our knowledge, this is the first description of a leptospiral protein exhibiting moonlighting activities.
